# Genome-Wide SNP Discovery and Population Genetic Analysis of *Mesocentrotus nudus* in China Seas

**DOI:** 10.3389/fgene.2021.717764

**Published:** 2021-08-19

**Authors:** Quanchao Wang, Ying Liu, Lang Yan, Linlin Chen, Baoquan Li

**Affiliations:** ^1^Key Laboratory of Coastal Biology and Bioresource Utilization, Yantai Institute of Coastal Zone Research, Chinese Academy of Sciences, Yantai, China; ^2^Center for Ocean Mega-Science, Chinese Academy of Sciences, Qingdao, China; ^3^University of Chinese Academy of Sciences, Beijing, China

**Keywords:** *Mesocentrotus nudus*, single-nucleotide polymorphism discovery, genetic diversity, population structure, China seas

## Abstract

*Mesocentrotus nudus* is an important commercially aquatic species because of its high edible and medicinal values. However, wild stocks have dramatically decreased in recent decades. Understanding the population structure and genetic diversity can provide vital information for genetic conservation and improvement. In the present study, the genotyping-by-sequencing (GBS) approach was adopted to identify the genome-wide single-nucleotide polymorphisms (SNPs) from a collection of 80 individuals consisting of five geographical populations (16 individuals from each population), covering the natural habitats of *M. nudus* in China seas. An average of 0.96-Gb clean reads per sample were sequenced, and a total of 51,738 biallelic SNPs were identified. Based on these SNPs, diversity index analysis showed that all populations have a similar pattern with positive F_is_ (0.136) and low Ne (724.3). Low genetic differentiation and high genetic connectivity among five geographical populations were detected by pairwise F_st_, principal component analysis (PCA), admixture, and phylogenetic analysis. Besides, two YWL individuals originating from an isolated ancestor may imply that there is a genetically differentiated population in the adjacent sea. Overall, the results showed that GBS is an effective method to detect genome-wide SNPs for *M. nudus* and suggested that the protective measures and the investigation with larger spatial scale and sample size for *M. nudus* should be carried out in the future.

## Introduction

Sea urchin is an ancient group of marine invertebrates that belongs to the class of Echinoidea under phylum of Echinodermata. To date, about 850 species of sea urchins have been found worldwide (Harris and Eddy, 2015). *Mesocentrotus nudus*, a member of the family Strongylocentrotidae, inhabits the intertidal and subtidal rocky sea bottoms along the coast of northwestern Pacific (Agatsuma, 2020). *M. nudus* is a well-known edible sea urchin for its good taste and redundant nutrition of vitamins and unsaturated fatty acids in the gonad (Mi et al., 2014). Furthermore, sea urchin extracts have extensive biological effects, such as anticancer, antioxidant, antileukemia, anti-fatigue, and anti-inflammatory effects (Liu et al., 2007).

The high edible and medicinal values of *M. nudus* have greatly stimulated the consumption demand, which has led to serious overfishing of wild sea urchins (Andrew et al., 2002; Li and Qi, 2008). In addition, the natural habitat of *M. nudus*, mostly adjacent to the densely populated areas (Adachi et al., 2020), has also been heavily disturbed by human activities including land-sourced pollution, aquaculture pollution, and sea reclamation (Lukyanova et al., 2017; Song and Duan, 2019). Under the influence of overfishing and natural habitat loss, the wild population resources of *M. nudus* are declining rapidly in recent years (Andrew et al., 2002). Especially in China, *M. nudus* has been considered as an endangered species. Although the breeding of *M. nudus* has been implemented to solve the shortage problem of natural resource in China since the 1980s (Ding et al., 2007), the parents of the seedlings were mainly from the wild populations. Therefore, understanding the genetic attributes of wild populations of *M. nudus* not only provide basic information for resources assessment and management and but also can lay a foundation for germplasm improvement.

Molecular markers have become the preferred tools for population genetic analysis. However, previously, quite limited genetic information on molecular markers (e.g., AFLP, mtDNA, and SSR) for *M. nudus* were available; and the level and pattern of population genetic diversity and structure are still poorly understood (Zhang et al., 2012). Recently, advances in next-generation sequencing (NGS) have greatly reduced the cost of nucleotide sequencing and made the genetic marker discovery more convenient. Genotyping-by-sequencing (GBS) as a genomic approach can explore the data covering the whole genome range with reduced cost and also provide more high-resolution genetic information for species lacking genomic information (Elshire et al., 2011). So far, GBS has been widely applied to assess genetic diversity on marine organisms, e.g., *Ostrea lurida* (Silliman, 2019), *Sillago japonica* (Yang et al., 2020), and *Haliotis discus* (Wells and Dale, 2018). However, no report on the population genetic analysis of *M. nudus* by using GBS was found up to now.

In the present study, GBS was used to genotype a total of 80 individuals consisting of five populations, covering the distribution range of *M. nudus* in China seas. The objectives were to detect and genotype single-nucleotide polymorphisms (SNPs) at a genome-wide level and to characterize the genetic diversity and population structure of *M. nudus*. The results will provide useful information for the utility of GBS on the genetic analysis of sea urchin species and contribute to the population conservation and genetic improvement of *M. nudus*.

## Materials and Methods

### Sampling

**FIGURE 1:**
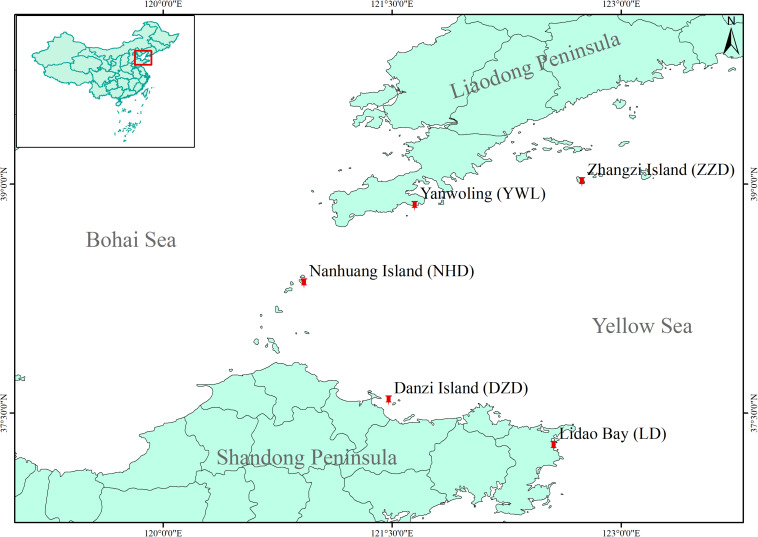
Map for sample locations of *Mesocentrotus nudus* in China.

In 2020, a total of 80 individuals were collected from five localities by SCUBA diving (16 individuals from each locality, Figure 1), covering the natural distribution range of *M. nudus* in China. Gonad tissue was sampled and fixed by ethanol on site. Genomic DNA of each sample was isolated from gonad tissue by using Plant Genomic DNA Kit (TIANGEN, Beijing, China) following the manual instruction. The purity and integrity of extracted DNA were determined by using a NanoDrop 1000 Spectrophotometer (NanoDrop, Wilmington, DE, United States) and electrophoresis on 1% agarose gel. Qualified genomic DNA was stored at −20°C.

### Sequencing and Genotyping

All samples were sequenced by using the GBS (Qi et al., 2018) method, which was performed by OE Biotech Co., Ltd. (Shanghai, China). Briefly, extracted DNA from each individual was digested with restriction enzymes *Pst*I-HF and *Msp*I, and the barcoded adapters and common adapter were respectively ligated on the *Pst*I cut site and the *Msp*I cut site of all samples by T4 DNA ligase. Then, the fragments of 300–700 bp were retrieved using recovery system of improved magnetic bead and amplified by PCR using high-fidelity enzymes. Finally, amplification fragments were sequenced on an Illumina Nova, PE150 (Illumina, Inc., San Diego, CA, United States). The generated raw reads were filtered to remove the potential adaptor sequences by using STACKS software (Catchen et al., 2013). After quality control, there was no reference genome available for aligning the clean reads for SNP discovery. Therefore, ustacks (-M 3), cstacks (-n 3), sstacks, tsv2bam, and gstacks programs using STACKS pipeline were used for *de novo* SNP discovery. The initial SNPs were filtered according to the following parameters: (1) minimum sequence depth was above 5; (2) the allelic number was equal to 2; (3) minor allele frequency (MAF) was greater than 0.05, and (4) missing rate was lower than 10%. The quality control of SNP data was performed using vcftools (Danecek et al., 2011) and R software (R Core Team, 2018).

### Genetic Diversity Analysis

Population genetic diversity within overall populations and within each of the five populations was assessed. The polymorphic information content (PIC) was calculated using R package snpReady (Granato et al., 2018). The observed heterozygosity (Ho), expected heterozygosity (He), and inbreeding coefficient (F_is_) were calculated at each SNP locus, and also across all loci, using hierfstat package in R software (Goudet, 2005). Besides, multilocus heterozygosity (MLH) and standardized MLH (sMLH) were calculated for all individuals using inbreedR package (Stoffel et al., 2016). The effective population size (Ne) using the linkage disequilibrium (LD) method was estimated by using NeEstimator V2.01 (Do et al., 2014). Considering the small size of each population, the Ne was only analyzed for overall populations.

### Population Structure Analysis

Initial analysis of population structure was carried out by principal component analysis (PCA) using R package SNPRelate (Zheng et al., 2012) and plotted using Scatterplot3d package (Ligges and Mächler, 2002). In addition, the levels of ancestry and admixture proportions of individuals were assessed by using R package LEA (Frichot and François, 2015). The number of ancestral populations (k) ranging between 1 and 10 were tested, and 10 replicate analyses were run for each value of k. The optimal k was chosen based on cross-entropy and cross-validation errors (Frichot et al., 2014). To better understand fine-scale patterns of genetic structure between populations, pairwise F_st_ values were calculated with the function pairwise.neifst and significance was tested using 1,000 bootstrap replicates with the function boot.ppfst in the R package hierfstat (Goudet, 2005).

### Phylogenetic Analysis

The individual-level phylogenetic tree was constructed using SNPhylo (Lee et al., 2014) with an automated bash shell script snphylo.sh. For this analysis, an LD threshold (r^2^ = 0.8) was used to reduce SNP redundancy, and 1,000 bootstrap analyses were fulfilled. The individual phylogenetic tree was visualized by using R package ggtree (Yu et al., 2017). Besides, the population-level phylogeny using the maximum likelihood approach was also inferred by using TreeMix (Pickrell and Pritchard, 2012). Considering that the missing data can affect the accuracy of TreeMix inference, the SNPs with missing values were removed. The population-level phylogeny was drawn by using plotting_funcs.R script^1^.

## Results

### Sequencing and Genotyping

All samples were sequenced by using GBS at efficient sequencing depths of approximately 34.11-fold. In total, 76.76 Gb of raw data was produced, with an average of 0.96 Gb per individual. After filtering, a total of 72.39 Gb of clean data was retained with an average effective rate (clean reads compared with raw reads) of 94.30%. Statistics on clean reads further showed the quality value 20 (Q20) ≥ 96.33% and quality value 30 (Q30) ≥ 90.41%, and the guanine–cytosine (GC) contents ranged from 40.43 to 42.07%. The reference sequence constructed using STACKS pipeline contained 439,316 tags with an average length of 277 bp, the longest one being 1,036 bp and the shortest one being 71 bp. Initially, 992,728 putative SNPs were identified. After quality control based on successive filtering steps for sequence depth, allelic number, MAF, and missing rate, a total of 51,738 biallelic SNP markers located on 15,562 tags were retained.

### Genetic Diversity

Several diversity indices are shown in Table 1. The PIC ranged from 0.213 (LD) to 0.220 (DZD), with an average of 0.217. The observed heterozygosity (Ho) and expected heterozygosity (He) were in the ranges Ho = 0.230 to 0.239 and He = 0.266 to 0.276, with an average of Ho = 0.235 and He = 0.272, respectively. Regarding the inbreeding coefficient (F_is_), the lowest value was detected in the YWL and the highest in ZZD. Average individual MLH ranged from 0.241 to 0.246. Average sMLH values slightly varied across populations. The estimated effective population size (Ne) was 724.3 for all populations.

**TABLE 1:** Genetic diversity summary of *Mesocentrotus nudus* from five localities in China.

	PIC	Ho	He	F_is_	MLH	sMLH	Ne
DZD	0.220	0.239	0.276	0.135	0.246	1.005	–
NHD	0.219	0.238	0.275	0.135	0.244	1.000	–
LD	0.213	0.230	0.266	0.135	0.244	1.006	–
ZZD	0.215	0.232	0.270	0.141	0.241	0.988	–
YWL	0.217	0.236	0.273	0.133	0.246	1.004	–
ALL	0.217	0.235	0.272	0.136	0.244	1.001	724.3

*PIC, polymorphism information content; Ho, observed heterozygosity; He, expected heterozygosity; F_*is*_, inbreeding coefficient; MLH, multilocus heterozygosity; sMLH, standardized MLH; Ne, effective population size.*

### Population Genetic Structure

**FIGURE 2:**
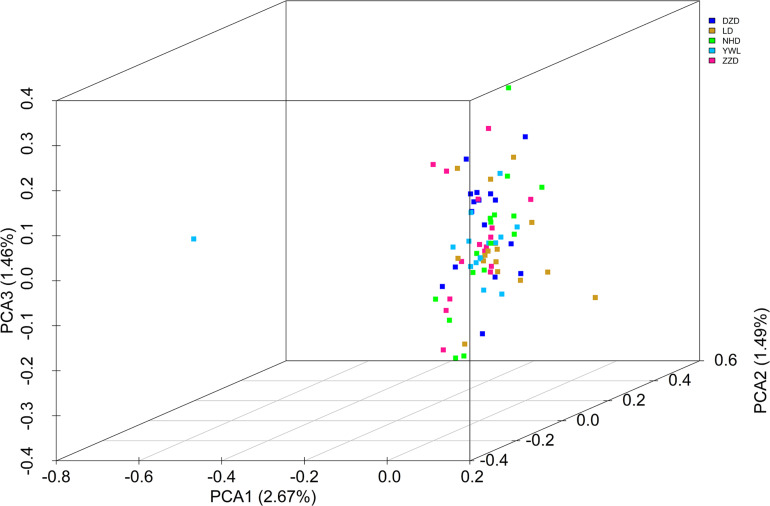
Principal components analysis (PCA) 3D plot of the 80 individuals using genome-wide single-nucleotide polymorphisms (SNPs).

**FIGURE 3:**
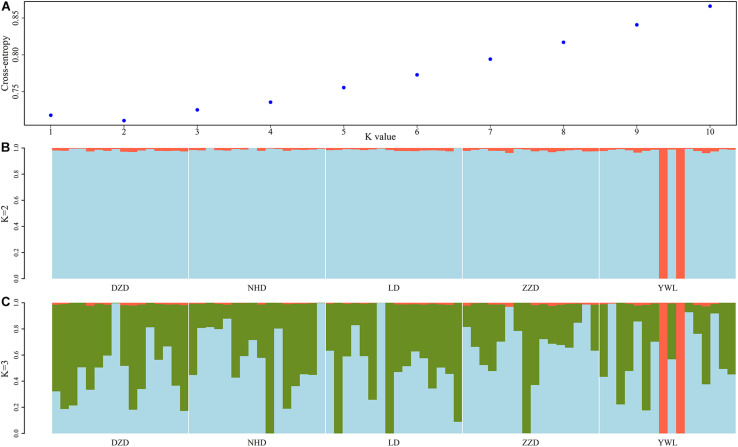
Admixture analyses of 80 individuals based on the genome-wide single-nucleotide polymorphisms (SNPs). **(A)** Cross-validation plot for the number of ancestral populations (k). **(B)** Stacked bar plot for the k = 2. **(C)** Stacked bar plot for the k = 3. Each column represents an individual, and the length of colored segments denotes the proportion of an individual’s genome inherited from one of k ancestral populations.

The PCA revealed that the majority of samples were clustered together, and no geographical patterns were observed (Figure 2). Similar results can be achieved from the admixture analysis (Figure 3). With the use of LEA, the minimum value of the cross-validation errors was 0.710 when k = 2, and the values continuously increased with k from 3 to 10 (Figure 3A). All individuals were classified into two groups at the level of k = 2, but one group contained only two individuals from YWL (Figure 3B). To further investigate the population structure, the analysis at k = 3 was also performed, and similar results were observed (Figure 3C). The pairwise F_st_ values ranged from 0.0001 to 0.0039 with a mean value of 0.0019 (Table 2). The highest level of differentiation was observed between LD and YWL, whereas DZD and NHD differentiated the lowest.

**TABLE 2:** Pairwise F_st_ values among populations.

	DZD	NHD	LD	ZZD	YWL
DZD	–	–	–	–	–
NHD	0.0001	–	–	–	–
LD	0.0014	0.0018	–	–	–
ZZD	0.0003	0.0016	0.0012	–	–
YWL	0.0019	0.0032	0.0039	0.0031	–

*No significant F_*st*_ values were found at *p* < 0.05.*

### Phylogenetic Tree

**FIGURE 4:**
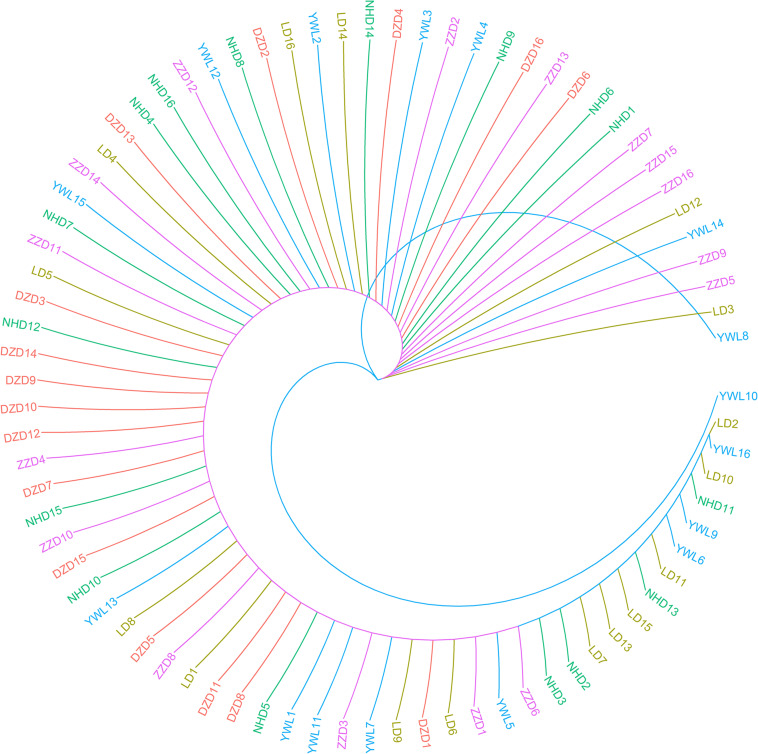
Individual-level phylogeny constructed using SNPhylo.

**FIGURE 5:**
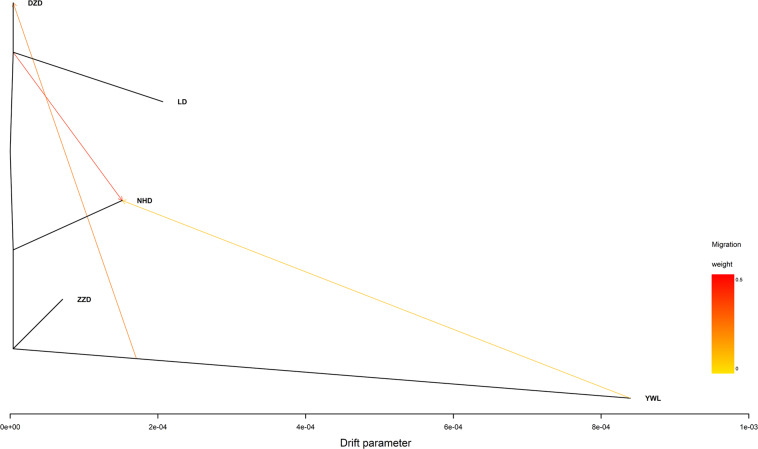
Population-level phylogeny with migration edges constructed using TreeMix.

The individual-level phylogenetic tree was constructed with 33,178 biallelic SNPs (Figure 4), in which all individuals belong to the same branch except YWL8 and YWL10. The population-level phylogeny constructed with 5,460 biallelic SNPs showed that the five populations appeared to originate from the same group, and three migration events were modeled among different branches (Figure 5). Overall, the phylogenetic tree analysis coincided with the PCA and admixture analysis, which corroborated a high genetic connectivity with even biological mixing among all locations.

## Discussion

Reduced-representation sequencing could provide an opportunity for those species without prior genetic resources to understand their basic properties of the genome (Silliman, 2019; Yang et al., 2020). In this study, a large number of genome fragments were obtained for *M. nudus* by using GBS. By analyzing the DNA base composition, the percentage of GC content was up to 41%, which was higher than that of the reported sea urchin, including *Strongylocentrotus purpuratus* (36.9%) (Sodergren et al., 2006), and *Lytechinus variegatus* (36.1%) (Davidson et al., 2020). Considering that genomic GC content is closely associated with genome size and significantly affects the genome functioning and species ecology (Šmarda et al., 2014), this result suggested that *M. nudus* may have a more complex genome than *S. purpuratus* and *L. variegatus*.

This study is the first report to identify genome-wide SNPs for *M. nudus* and to determine the genetic diversity and population structure of this species in China seas. Several indices concerning heterozygosity were firstly analyzed across five geographical populations. In general, the DZD population displayed the highest genetic diversity. This result may benefit from the good ecological status in the surrounding waters of Danzi island especially after removal of the marine raft culture and minimizing the amount of waste water discharged into the coastal waters since 2010 (Li et al., 2013). In contrast, the Lidao Bay as an important fishery and aquaculture water was seriously affected by anthropogenic disturbance (Jiang et al., 2013; Wu et al., 2016), which may result in the lowest PIC, Ho, and He for LD population. Besides, compared with previous studies on the population genetics of aquatic animals by using genome-wide SNPs, a moderate level of heterozygosity was observed for *M. nudus* in the current study. For example, the average Ho in *M. nudus* was lower than that in *Oncorhynchus keta* (Waples et al., 2017) and *Paracentrotus lividus* (Carreras et al., 2020), but higher than in several species such as *Oreochromis urolepis* (Nyinondi et al., 2020), *O. lurida* (Silliman, 2019), and *Isocladus armatus* (Wells and Dale, 2018). However, positive F_is_ values have been observed in *M. nudus*. F_is_ values could reflect the deviation from the Hardy–Weinberg equilibrium (HWE) genotype frequencies and indirectly reflect relative population heterozygosity. Generally, positive F_is_ values may be caused by mating among relatives, life history trait with free-spawned planktonic sperm, and selection acting on the genetic markers (Zouros and Foltz, 1984; David et al., 1997; Whitaker, 2004; Addison and Hart, 2005). In the current study, the estimated Ne value for *M. nudus* was significantly lower than that for other marine species such as *Chinook salmon* (Larson et al., 2014), *Salmo salar* (Johnston et al., 2014), and *Panulirus homarus* (Al-Breiki et al., 2018). According to the proposal by Frankham et al. (2014), this value is too low to retain evolutionary potential for *M. nudus* and thus may lead to inbreeding. Besides, the life history of *M. nudus* can also contribute to the positive F_is_ because the species like *M. nudus* with free-spawned planktonic sperm tend to have higher F_is_ than those species that copulate or have some form of direct sperm transfer to females or benthic egg masses (Addison and Hart, 2005). In addition, the population were sampled along coastal zone of the Yellow Sea and Bohai Sea, where they have been heavily disturbed by anthropogenic activities including land-source pollution, aquaculture pollution, and reclamation. The phenomena of eutrophication and hypoxia are happening with an increasing frequency in recent years, resulting in drastic changes in the physical and chemical parameters of seawater such as nutrient content, dissolved oxygen (DO), and pH especially in a small spatial scale. These drastic and persistent environmental disturbances can not only lead to the reduction of population size but also impose strong selective pressure on *M. nudus*, which is relatively weak in activity and thus may contribute to the high F_is_ value. Overall, the positive F_is_ and low Ne suggested that the genetic diversity of *M. nudus* should be constantly monitored, and the protective measures should be carried out in China.

In order to assess the genetic differences among populations, pairwise F_st_ values were estimated. The results showed that all F_st_ values were far less than 0.05, which implies that there were low genetic differentiation and high genetic connectivity among five geographical populations. This result can be confirmed by PCA, admixture analysis, and phylogenetic analysis. This phenomenon may be mainly related to the lack of dispersal barriers in the Yellow Sea and Bohai Sea, and also the fact that *M. nudus* has a pelagic phase lasting for 1 month in the water column (Dautov et al., 2020). However, it is worth noting that two individuals from YWL deviated significantly from the main cluster and have little genetic association with other individuals. This phenomenon can also be found in similar studies. For example, individual ancestry analysis for five populations of Pacific cod showed that two individuals from coastal area had no similar ancestries as coastal fish (Drinan et al., 2018). Interestingly, these individuals displayed the same ancestry as the fish from the Salish Sea. Similarly, some seahorses from New Zealand were descendants of Australian ancestors (Ashe and Wilson, 2019). Therefore, in the current study, two differentiated individuals from YWL may imply that there is a genetically differentiated population in the adjacent sea. Especially, it is worth exploring whether the population structure of *M. nudus* from the Japan Sea will be different from that of the Yellow Sea and Bohai Sea, considering the two sea areas are separated by the Korean Peninsula, which might form a dispersal barrier. Besides, the small sampling size in the current study may be a potential factor leading to the cryptic genetic structure of *M. nudus* that may not be fully revealed. Therefore, the investigation with larger spatial scale and sample size of *M. nudus* needs to be implemented to fully assess the population structure and genetic diversity of *M. nudus* in the future.

## Conclusion

In the present study, a total of 51,738 high-quality SNPs were detected, indicating that the GBS technology is a powerful tool for the genome-wide SNP discovery of *M. nudus*. Diversity index analysis showed that all populations have a similar pattern with positive F_is_ and low Ne, suggesting that it is necessary to carry out the conservation of *M. nudus* in China. Low genetic differentiation and high genetic connectivity among five geographical populations were detected by pairwise F_st_, PCA, admixture analysis, and phylogenetic analysis. Besides, two individuals originating from an isolated ancestor were identified, which may be crucial for the conservation of genetic diversity and germplasm improvement. Thus, the investigation with larger spatial scale and sample size needs to be implemented to assess the population structure and genetic diversity of *M. nudus* in the future.

## Data Availability Statement

The datasets presented in this study can be found in online repositories. The names of the repository/repositories and accession number(s) can be found below: GenBank (Accession number: PRJNA738544).

## Ethics Statement

Each of the procedures that were used to handle and treat the *Mesocentrotus nudus* during this study was approved by the Key Laboratory of Coastal Biology and Biological Resources Utilization, Yantai Institute of Coastal Zone Research, Chinese Academy of Sciences, prior to the initiation of the study and the Animal Management Regulations, revised on March 1, 2017, No. 676.

## Author Contributions

QW conducted the experiment and data processing. BL conceived and supervised the project. QW and YL collected the experimental animals. LY and LC contributed to preparing the genomic DNA for SNP genotyping. QW and BL wrote the manuscript. All authors have read and approved the manuscript.

## Conflict of Interest

The authors declare that the research was conducted in the absence of any commercial or financial relationships that could be construed as a potential conflict of interest.

## Publisher’s Note

All claims expressed in this article are solely those of the authors and do not necessarily represent those of their affiliated organizations, or those of the publisher, the editors and the reviewers. Any product that may be evaluated in this article, or claim that may be made by its manufacturer, is not guaranteed or endorsed by the publisher.

## Footnotes

1
https://github.com/joepickrell/pophistory-tutorial/blob/master/example2/plotting_funcs.R

## References

[B1] Adachi K. Suzuki T. Okumura S. I. Funayama S. Moriyama S. (2020). Influence of the 2011 Tohoku tsunami on the genetic structure of wild sea urchin (*Mesocentrotus nudus*) populations in Sanriku, Japan. *Mar. Ecol.* 41:e12584. 10.1111/maec.12584

[B2] Addison J. Hart M. (2005). Spawning, copulation and inbreeding coefficients in marine invertebrates. *Biol. Lett.* 1 450–453. 10.1098/rsbl.2005.0353 17148230 PMC1626360

[B3] Agatsuma Y. (2020). *Mesocentrotus nudus*. *Dev. Aquac. Fish. Sci.* 43 627–641.

[B4] Al-Breiki R. D. Kjeldsen S. R. Afzal H. Al Hinai M. S. Zenger K. R. Jerry D. R. (2018). Genome-wide SNP analyses reveal high gene flow and signatures of local adaptation among the scalloped spiny lobster (*Panulirus homarus*) along the Omani coastline. *BMC Genomics* 19:690. 10.1186/s12864-018-5044-8 30231936 PMC6146514

[B5] Andrew N. Agatsuma Y. Ballesteros E. Bazhin A. Creaser E. Barnes D. (2002). Status and management of world sea urchin fisheries. *Oceanogr. Mar. Biol.* 40 343–425.

[B6] Ashe J. L. Wilson A. B. (2019). Navigating the southern seas with small fins: genetic connectivity of seahorses (*Hippocampus abdominalis*) across the Tasman Sea. *J. Biogeogr.* 47 207–219. 10.1111/jbi.13733

[B7] Carreras C. García-Cisneros A. Wangensteen O. S. Ordóñez V. Palacín C. Pascual M. (2020). East is east and west is west: population genomics and hierarchical analyses reveal genetic structure and adaptation footprints in the keystone species *Paracentrotus lividus* (Echinoidea). *Divers. Distrib.* 26 382–398. 10.1111/ddi.13016

[B8] Catchen J. Hohenlohe P. A. Bassham S. Amores A. Cresko W. A. (2013). Stacks: an analysis tool set for population genomics. *Mol. Ecol.* 22 3124–3140. 10.1111/mec.12354 23701397 PMC3936987

[B9] Danecek P. Auton A. Abecasis G. Albers C. A. Banks E. DePristo M. A. (2011). The variant call format and VCFtools. *Bioinformatics* 27 2156–2158. 10.1093/bioinformatics/btr330 21653522 PMC3137218

[B10] Dautov S. Dautova T. Kashenko S. (2020). Towards a scientific-based farming of sea urchins: first steps in the cultivation of *Diadema setosum*, *Diadema savignyi* and *Mesocentrotus nudus*. *APN Sci. Bull.* 10 109–118. 10.30852/sb.2020.1284

[B11] David P. Perdieu M. A. Pernot A. F. O. Jarne P. (1997). Fine-grained spatial and temporal population genetic structure in the marine bivalve *Spisula ovalis*. *Evolution* 51 1318–1322. 10.2307/2411061 28565480

[B12] Davidson P. L. Guo H. Wang L. Berrio A. Zhang H. Chang Y. (2020). Chromosomal-level genome assembly of the sea urchin *Lytechinus variegatus* substantially improves functional genomic analyses. *Genome Biol. Evol.* 12 1080–1086. 10.1093/gbe/evaa101 32433766 PMC7455304

[B13] Ding J. Chang Y. Wang C. Cao X. (2007). Evaluation of the growth and heterosis of hybrids among three commercially important sea urchins in China: *Strongylocentrotus nudus*, *S. intermedius* and *Anthocidaris crassispina*. *Aquaculture* 272 273–280. 10.1016/j.aquaculture.2007.07.231

[B14] Do C. Waples R. S. Peel D. Macbeth G. Tillett B. J. Ovenden J. R. (2014). NeEstimator v2: re-implementation of software for the estimation of contemporary effective population size (Ne) from genetic data. *Mol. Ecol. Resour.* 14 209–214. 10.1111/1755-0998.12157 23992227

[B15] Drinan D. P. Gruenthal K. M. Canino M. F. Lowry D. Fisher M. C. Hauser L. (2018). Population assignment and local adaptation along an isolation-by-distance gradient in Pacific cod (*Gadus macrocephalus*). *Evol. Appl.* 11 1448–1464. 10.1111/eva.12639 30151052 PMC6100185

[B16] Elshire R. J. Glaubitz J. C. Sun Q. Poland J. A. Kawamoto K. Buckler E. S. (2011). A robust, simple genotyping-by-sequencing (GBS) approach for high diversity species. *PloS One* 6:e19379. 10.1371/journal.pone.0019379 21573248 PMC3087801

[B17] Frankham R. Bradshaw C. J. A. Brook B. W. (2014). Genetics in conservation management: revised recommendations for the 50/500 rules, red list criteria and population viability analyses. *Biol. Conserv.* 170 56–63. 10.1016/j.biocon.2013.12.036

[B18] Frichot E. François O. (2015). LEA: an R package for landscape and ecological association studies. *Methods Ecol. Evol.* 6 925–929. 10.1111/2041-210X.12382

[B19] Frichot E. Mathieu F. Trouillon T. Bouchard G. François O. (2014). Fast and efficient estimation of individual ancestry coefficients. *Genetics* 196 973–983. 10.1534/genetics.113.160572 24496008 PMC3982712

[B20] Goudet J. (2005). Hierfstat, a package for R to compute and test hierarchical F-statistics. *Mol. Ecol. Notes* 5 184–186. 10.1111/j.1471-8286.2004.00828.x

[B21] Granato I. S. C. Galli G. de Oliveira Couto E. G. e Souza M. B. Mendonça L. F. Fritsche-Neto R. (2018). Snpready: a tool to assist breeders in genomic analysis. *Mol. Breed.* 38:102. 10.1007/s11032-018-0844-8

[B22] Harris L. G. Eddy S. D. (2015). “Sea urchin ecology and biology,” in *Echinoderm Aquaculture*, eds Brown N. P. Eddy S. D. (Hoboken, NJ: John Wiley & Sons), 3–24.

[B23] Jiang Z. Fang J. Mao Y. Han T. Wang G. (2013). Influence of seaweed aquaculture on marine inorganic carbon dynamics and sea-air CO2 flux. *J. World Aquac. Soc.* 44 133–140. 10.1111/jwas.12000

[B24] Johnston S. E. Orell P. Pritchard V. L. Kent M. P. Lien S. Niemelä E. (2014). Genome-wide SNP analysis reveals a genetic basis for sea-age variation in a wild population of Atlantic salmon (*Salmo salar*). *Mole. Ecol.* 23 3452–3468. 10.1111/mec.12832 24931807

[B25] Larson W. A. Seeb L. W. Everett M. V. Waples R. K. Templin W. D. Seeb J. E. (2014). Genotyping by sequencing resolves shallow population structure to inform conservation of Chinook salmon (*Oncorhynchus tshawytscha*). *Evol. Appl.* 7 355–369. 10.1111/eva.12128 24665338 PMC3962296

[B26] Lee T.-H. Guo H. Wang X. Kim C. Paterson A. H. (2014). SNPhylo: a pipeline to construct a phylogenetic tree from huge SNP data. *BMC Genomics* 15:162. 10.1186/1471-2164-15-162 24571581 PMC3945939

[B27] Li B. Wang Q. Li B. (2013). Assessing the benthic ecological status in the stressed coastal waters of Yantai, Yellow Sea, using AMBI and M-AMBI. *Mar. Pollut. Bull.* 75 53–61. 10.1016/j.marpolbul.2013.08.007 23993073

[B28] Li J. Qi L. (2008). A set of microsatellite markers for use in the endangered sea urchin *Strongylocentrotus nudus* developed from *S. purpuratus* ESTs. *Conserv. Genet.* 9 743–745. 10.1007/s10592-007-9382-3

[B29] Ligges U. Mächler M. (2002). Scatterplot3d-an r package for visualizing multivariate data. *J. Statist. Softw.* 8 1–20.

[B30] Liu C. Lin Q. Yi G. Liang Y. Xing Y. Tao X. (2007). Characterization and antitumor activity of a polysaccharide from *Strongylocentrotus nudus* eggs. *Carbohydr. Polym.* 67 313–318. 10.1016/j.carbpol.2006.05.024

[B31] Lukyanova O. N. Zhuravel E. V. Chulchekov D. N. Mazur A. A. (2017). Sea urchin embryogenesis as bioindicators of marine pollution in impact areas of the Sea of Japan/East Sea and the Sea of Okhotsk. *Arch. Environ. Contam. Toxicol.* 73 322–333. 10.1007/s00244-017-0388-7 28528417

[B32] Mi X. Wei Z. Zhou Z. Liu X. (2014). Identification and profiling of sex-biased microRNAs from sea urchin *Strongylocentrotus nudus* gonad by solexa deep sequencing. *Comp. Biochem. Physiol. Part D Genom. Proteomics* 10 1–8. 10.1016/j.cbd.2014.01.001 24486540

[B33] Nyinondi C. S. Mtolera M. S. Mmochi A. J. Lopes Pinto F. A. Houston R. D. de Koning D. J. (2020). Assessing the genetic diversity of farmed and wild Rufiji tilapia (*Oreochromis urolepis urolepis*) populations using ddRAD sequencing. *Ecol. Evol.* 10 10044–10056. 10.1002/ece3.6664 33005362 PMC7520224

[B34] Pickrell J. Pritchard J. (2012). Inference of population splits and mixtures from genome-wide allele frequency data. *PLoS Genet.* 8:e1002967. 10.1038/npre.2012.6956.1 23166502 PMC3499260

[B35] Qi P. Gimode D. Saha D. Schröder S. Chakraborty D. Wang X. (2018). UGbS-Flex, a novel bioinformatics pipeline for imputation-free SNP discovery in polyploids without a reference genome: finger millet as a case study. *BMC Plant Biol.* 18:117. 10.1186/s12870-018-1316-3 29902967 PMC6003085

[B36] R Core Team (2018). *R: A Language and Environment for Statistical Computing. Computing.* Vienna: R Core Team.

[B37] Silliman K. (2019). Population structure, genetic connectivity, and adaptation in the *Olympia oyster* (*Ostrea lurida*) along the west coast of North America. *Evol. Appl.* 12 923–939. 10.1111/eva.12766 31080505 PMC6503834

[B38] Šmarda P. Bureš P. Horová L. Leitch I. J. Mucina L. Pacini E. (2014). Ecological and evolutionary significance of genomic GC content diversity in monocots. *Proc. Natl. Acad. Sci. U.S.A.* 111 E4096–E4102. 10.1073/pnas.1321152111 25225383 PMC4191780

[B39] Sodergren E. Weinstock G. M. Davidson E. H. Cameron R. A. Gibbs R. A. Angerer R. C. (2006). The genome of the sea urchin *Strongylocentrotus purpuratus*. *Science* 314 941–952. 10.1126/science.1133609 17095691 PMC3159423

[B40] Song J. Duan L. (2019). “The bohai sea,” in *World Seas: An Environmental Evaluation*, 2nd Edn, ed. Sheppard C. (Cambridge, MA: Academic Press), 377–394.

[B41] Stoffel M. A. Esser M. Kardos M. Humble E. Nichols H. David P. (2016). Inbreedr: an R package for the analysis of inbreeding based on genetic markers. *Methods Ecol. Evol.* 7 1331–1339. 10.1111/2041-210X.12588

[B42] Waples R. Seeb J. Seeb L. (2017). Congruent population structure across paralogous and nonparalogous loci in Salish sea chum salmon (*Oncorhynchus keta*). *Mol. Ecol.* 26 4131–4144. 10.1111/mec.14163 28452089

[B43] Wells S. J. Dale J. (2018). Contrasting gene flow at different spatial scales revealed by genotyping-by-sequencing in *Isocladus armatus*, a massively colour polymorphic New Zealand marine isopod. *PeerJ* 6:e5462. 10.7717/peerj.5462 30155361 PMC6109376

[B44] Whitaker K. (2004). Non-random mating and population genetic subdivision of two broadcasting corals at Ningaloo Reef. Western Australia. *Mar. Biol.* 144 593–603. 10.1007/s00227-003-1220-7

[B45] Wu Z. Zhang X. Lozano-Montes H. M. Loneragan N. R. (2016). Trophic flows, kelp culture and fisheries in the marine ecosystem of an artificial reef zone in the Yellow Sea. *Estuar. Coast. Shelf Sci.* 182 86–97. 10.1016/j.ecss.2016.08.021

[B46] Yang T. Gao T. Meng W. Jiang Y. (2020). Genome-wide population structure and genetic diversity of Japanese whiting (*Sillago japonica*) inferred from genotyping-by-sequencing (GBS): implications for fisheries management. *Fish. Res.* 225:105501. 10.1016/j.fishres.2020.105501

[B47] Yu G. Smith D. K. Zhu H. Guan Y. Lam T. T. Y. (2017). Ggtree: an R package for visualization and annotation of phylogenetic trees with their covariates and other associated data. *Methods Ecol. Evol.* 8 28–36. 10.1111/2041-210X.12628

[B48] Zhang L. Dong Y. Yang A. Chen Z. Wang X. Guan X. (2012). Genetic diversity analysis of three geographic populations of sea urchin *Strongylocentrotus nudus* by AFLP. *Fish. Sci.* 31 132–136. 10.16378/j.cnki.1003-1111.2012.03.007

[B49] Zheng X. Levine D. Shen J. Gogarten S. M. Laurie C. Weir B. S. (2012). A high-performance computing toolset for relatedness and principal component analysis of SNP data. *Bioinformatics* 28 3326–3328. 10.1093/bioinformatics/bts606 23060615 PMC3519454

[B50] Zouros E. Foltz D. W. (1984). Possible explanations of heterozygote deficiency in bivalve mollusks. *Malacologia* 25 583–591.

